# Vaginal yolk sac tumor resected by a novel laparo/endoscope-assisted posterior sagittal approach: a case report

**DOI:** 10.1186/s40792-022-01520-8

**Published:** 2022-08-29

**Authors:** Motohiro Kano, Ryoya Furugane, Keita Hogetsu, Yuji Yamada, Junnosuke Maniwa, Tamotsu Kobayashi, Naoki Hashizume, Teizaburo Mori, Eiichiro Watanabe, Masataka Takahashi, Akihiro Fujino, Yutaka Kanamori, Keita Terashima, Kimikazu Matsumoto, Akihiro Yoneda

**Affiliations:** 1 Division of Pediatric Surgery, Department of Pediatric Surgical Specialties, National Center for Child Health and Development, 2-10 Okura, Setagaya-Ku, Tokyo, Japan; 2 Department of Pediatric Surgery, Keio University School of Medicine, 35 Shinanomachi, Shinjuku-Ku, Tokyo, Japan; 3 Children’s Cancer Center, National Center for Child Health and Development, 2-10 Okura, Setagaya-Ku, Tokyo, Japan; 4 Division of Surgical Oncology, Children’s Cancer Center, National Center for Child Health and Development, 2-10 Okura, Setagaya-Ku, Tokyo, Japan

**Keywords:** Germ cell tumor, Vaginal tumor, Laparoscopic surgery, Posterior sagittal approach, Yolk sac tumor, Case report

## Abstract

**Background:**

Yolk sac tumor (YST) is a germ cell tumor that is generally associated with good prognosis in children. It has been recently reported that vaginal YSTs can be cured using chemotherapy alone. Thus, minimal invasiveness and function preservation are pre-requisites for surgical approaches. Herein, we report a case of vaginal YST that was resected in a function-preserving manner using a unique combination of surgical approaches.

**Case presentation:**

In a 9-month-old Asian female infant, a vaginal tumor was detected while investigating for vaginal bleeding. The patient was referred to our hospital, and the tumor was diagnosed as a YST after incisional biopsy. Six courses of carboplatin-based chemotherapy were administered. Contrary to the findings in previous reports, the tumor was chemo-resistant and surgical resection was required for the residual tumor. During surgery, we utilized laparoscopic and endoscopic procedures to ensure tumor-free surgical margins at the cervix, rectum, and lateral wall of the vagina. Additionally, the posterior sagittal approach was used to easily resect the tumor, and the vagina was reconstructed leaving only inconspicuous scars in the intergluteal cleft. No complications occurred postoperatively. Pathological examination of the surgical specimen revealed tumor-free surgical margins. The patient received four cycles of intensified chemotherapy before and after the surgery. The patient has been disease-free for 6 months now.

**Conclusions:**

Our combination of laparo/endoscopic and posterior sagittal approach ensured a tumor-free macroscopic surgical margin with easier, cosmetically pleasing vaginal reconstruction, while preserving the anorectal and urinary functions. We believe that this approach could be utilized not only for vaginal YST, but also for any vaginal tumor, especially those arising from the posterior or lateral wall.

## Background

Yolk sac tumor (YST) is the most frequent type of extragonadal germ cell tumors in the female genital tract [1]. YST of the vagina occurs almost exclusively in the pediatric population (≤ 2 years) [2], and a differential diagnosis from rhabdomyosarcoma is essential. However, the diagnosis of YST is easier because it has a typical marker, alpha-fetoprotein (AFP). Serum AFP levels are elevated in most patients with YST [3].

The prognosis of malignant germ cell tumors in children is generally excellent, as 80% of the patients with extragonadal stage IV tumors survive [4]. Vaginal rhabdomyosarcoma is often the botryoid variant and has a good prognosis, similar to YST [5]. Furthermore, chemotherapy has drastically improved the prognosis of these tumors [6]; thus, a strategy to reduce the toxicity or side effects of treatment should be considered. In the treatment of rhabdomyosarcoma, secondary excision is only suggested when organ-preserving R0 resection can be achieved because it is usually sensitive to chemotherapy and irradiation [7]. Contrarily, the surgical strategy for the management of extracranial YST in pediatric patients is complete resection of the tumor before or after chemotherapy, as local control is critically important for patient survival [8]. Radiation therapy is generally ineffective in the treatment of YST. As the incidence of this tumor is rare, there is no standard approach for management of the tumor in other locations, except for the testis and ovary. Herein, we report a case of YST of the vagina completely resected using a combination of multiple approaches to preserve function and appearance.

## Case presentation

The patient was an Asian female infant with a family history of nephroblastoma (father) and retinoblastoma (sibling). Her perinatal status was normal, and her medical history was unremarkable. The patient’s mother accidentally observed vaginal bleeding in her 7-month-old daughter. Her previous doctor prescribed antibiotics; however, no improvement was observed. The patient was referred to a tertiary hospital where she was diagnosed with a vaginal tumor. She was then referred to our hospital at 9 months of age (day 0).

**Fig. 1:**
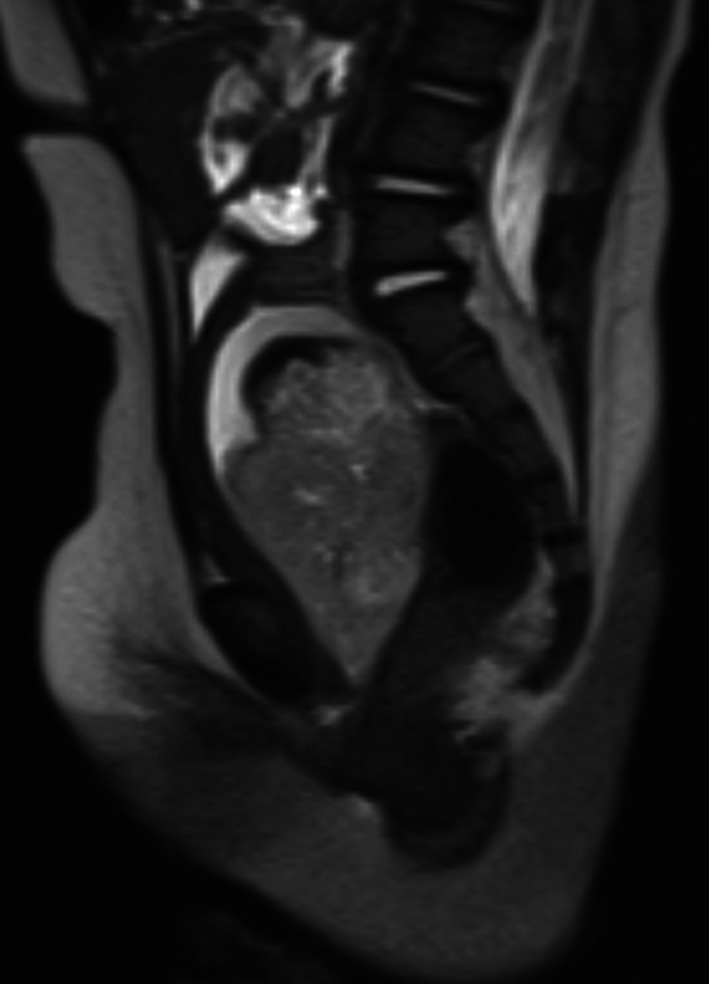
T2 sagittal image of the tumor at diagnosis. Magnetic resonance image shows a large solid mass in the vagina

Ultrasonography, enhanced computed tomography (CT), magnetic resonance imaging (MRI) (Fig. 1), and laboratory tests (including tumor markers) were performed. Distant or lymph node metastases were not detected. AFP was the sole abnormal tumor marker. The patient was clinically diagnosed with a YST arising from the vagina. A small incisional biopsy through the vaginal opening was performed on day 19 under general anesthesia, and the tumor was pathologically confirmed to be a YST with TNM staging T2bN0M0 and Children's Oncology Group (COG) staging III.

After her parents provided informed consent, she was treated with six courses of neoadjuvant JEB regimen, which included etoposide, bleomycin, and carboplatin from day 27. The preference of carboplatin over cisplatin was determined by her parents who wanted to minimize renal toxicities after the family history of pediatric cancers including kidney tumor.

**Fig. 2:**
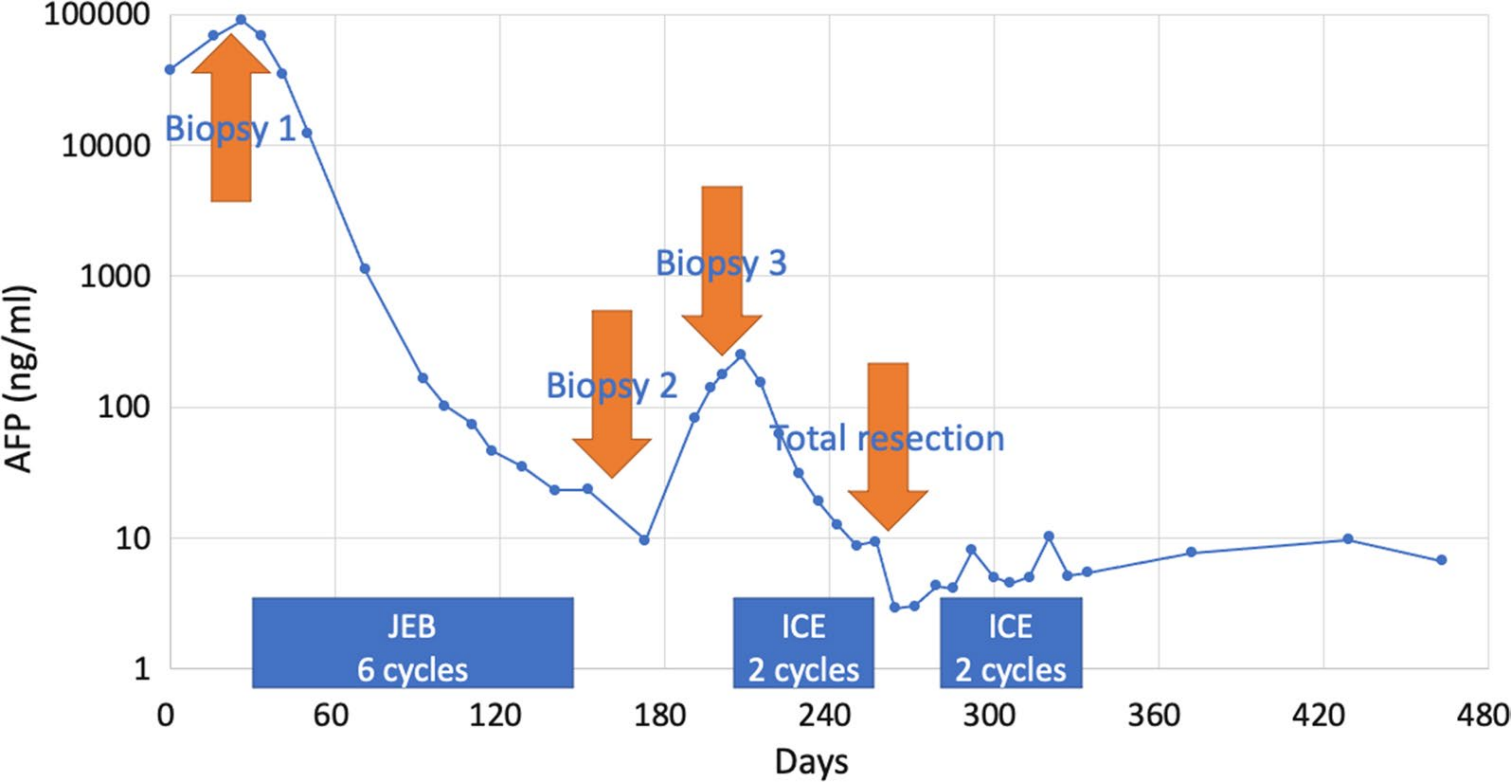
Trend of AFP level and treatment timeline. Day 0 was the patient’s first visit. The AFP values (ng/mL) are plotted on a logarithmic scale. JEB and ICE are the chemotherapy regimens. AFP, alpha-fetoprotein; JEB, carboplatin, etoposide, and bleomycin; ICE, ifosfamide, carboplatin, and etoposide

Four months after the primary chemotherapy, MRI revealed a shrunken, localized residual tumor. Her AFP declined logarithmically to 9.6 ng/mL on day 173 after completion of six cycles of JEB (cumulative agents per square: carboplatin 3600 mg, etoposide 2160 mg, and bleomycin 90 mg) (Fig. 2). Endoscopic evaluation and incisional biopsy were performed on day 156 revealing a wide-based yellowish tumor on the posterior vaginal wall. The pathology of the sample revealed approximately 10% viable tumor. After obtaining informed consent, anti-tumor treatment was completed, and the patient was followed under close observation with an expectation that the residual tumor undergoes differentiation and achieves remission.

**Fig. 3:**
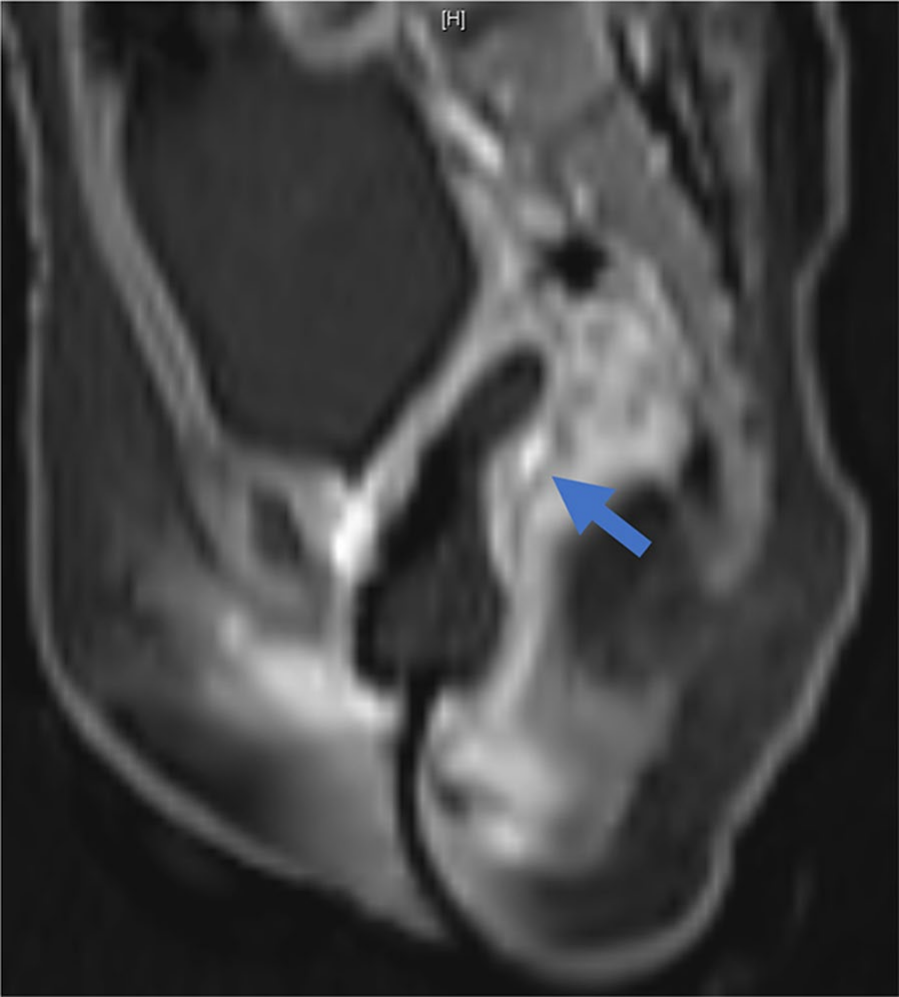
Gadolinium-enhanced magnetic resonance imaging of the tumor before total resection. Preoperative imaging reveals a residual tumor on the posterior wall of the vagina. The vagina is inflated with saline solution. Arrow: the tumor of interest

Nevertheless, there was an elevation in the AFP level after a month of observation (day 191) suggesting that the first-line chemotherapy was insufficient. Therefore, surgical intervention was recommended. Another incisional biopsy was performed on day 198, which confirmed the tumor viability. The ifosfamide, carboplatin, and etoposide (ICE) regimen was initiated as the second-line chemotherapy on day 201. After two cycles of ICE, the AFP declined to 8.7 ng/mL on day 250. CT and MRI revealed a tumor on the posterior wall, adjacent to the cervix (Fig. 3). There was neither distant nor lymph nodes metastasis, and the anterior wall of the rectum was free from direct tumor invasion. Since the tumor was chemotherapy-resistant, we decided on complete resection of the tumor after obtaining informed consent on day 261.

**Fig. 4:**
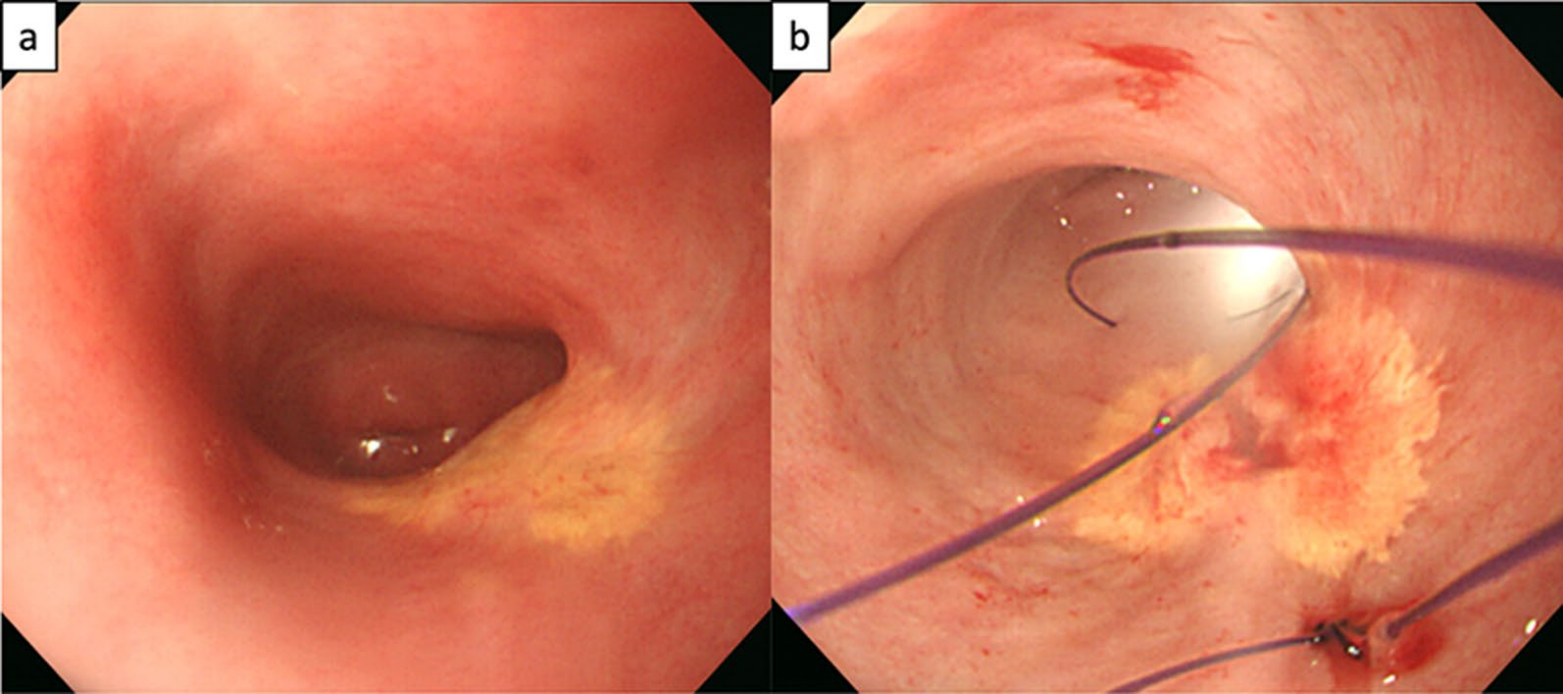
Endoscopic imaging of the vagina at the radical surgery. **a** Yellowish tumor resides on the left posterolateral wall of the vagina. **b** Marked sutures at the proximal and distal ends of the tumor. Light from the laparoscope is seen through the distal end of the vaginal wall

**Fig. 5:**
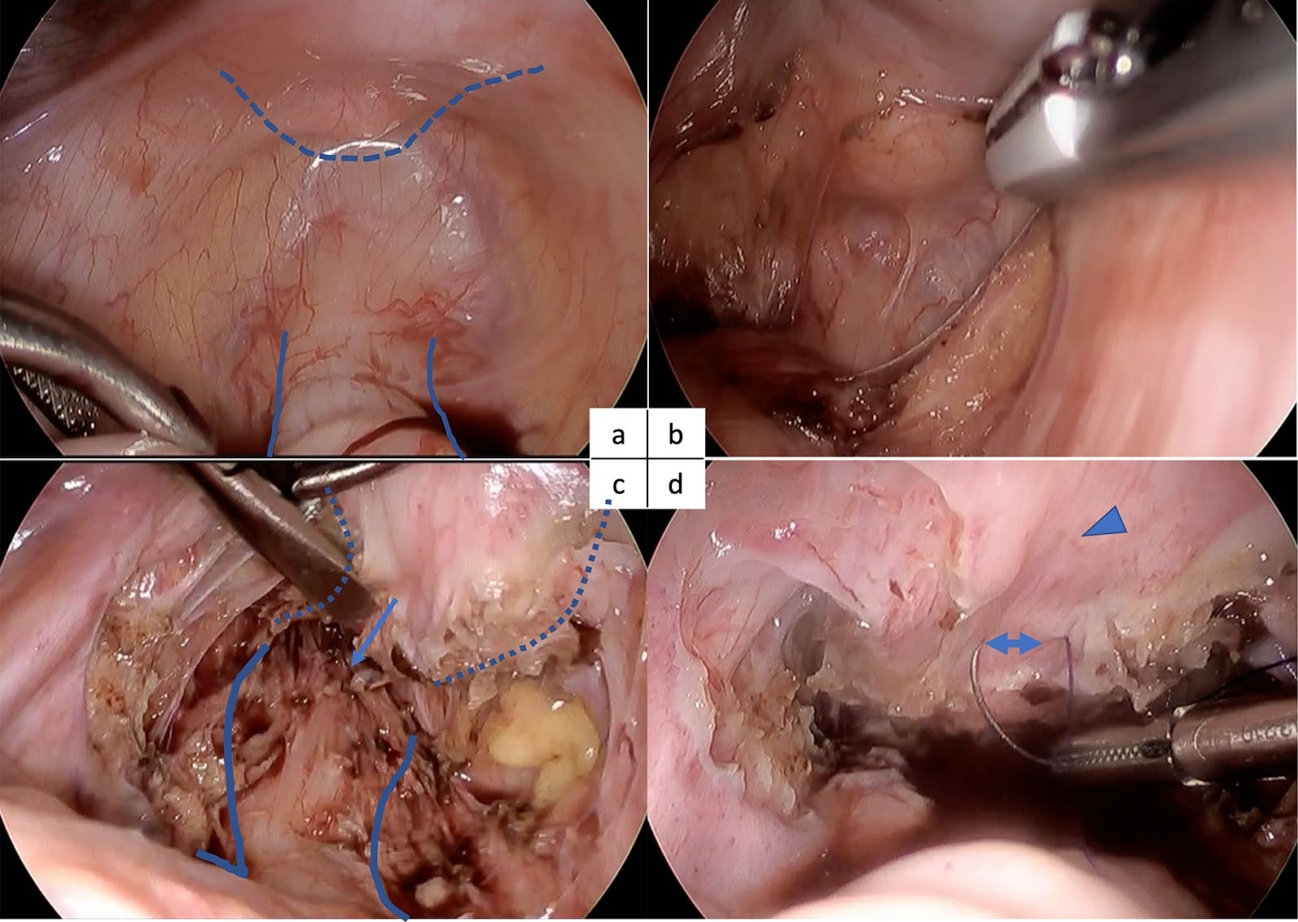
Laparoscopic view of the radical surgery. **a** Uterus (dashed line) and rectum (blue line) are exposed. **b** Separation between the vagina and rectum. **c** Ventral wall of the rectum (lined) is exposed and a marking suture (single arrow) for the distal end of the tumor is placed through the vagina (lined by dots). **d** A marked suture (double arrow) is visible at the proximal end of the tumor near the cervix (arrowhead)

Surgery began with vaginal evaluation using an Olympus GIF-PG260 endoscope (Olympus Corp., Tokyo, Japan). We confirmed that the tumor was confined to the posterior vaginal wall (Fig. 4a). The edge of the tumor was approximately 1 cm away from the cervix, and it was approximately 3 cm long and 3 cm wide, positioned from 3 o’clock on the left wall to 7 o’clock on the right wall. The patient was then fixed in the lithotomy position to secure the working space for intraoperative endoscopic investigation. A 5-mm cam port was inserted, and the intra-abdominal cavity was investigated. Laparoscopy confirmed the absence of dissemination of the tumor, nodules on the omentum, and swollen lymph nodes. A small amount of ascites was detected, but the cytology was negative. The top of the uterus was ligated to the abdominal wall to secure the space behind it. Laparoscopy provided good visualization of the area posterior to the vagina, and separation between the vagina and the anterior wall of the rectum was performed (Fig. 5a–d). During the laparoscopic procedure, the extent of the tumor was endoscopically confirmed, and marking sutures were placed on both proximal and distal ends of the tumor, penetrating through the vaginal wall to the pelvic cavity (Fig. 4b). Thus, the marker was identified laparoscopically, and the extent of the tumor was confirmed. No direct invasion of the tumor into the rectum was observed. The lateral side of the vagina was separated to mobilize the posterior half of the vagina.

**Fig. 6:**
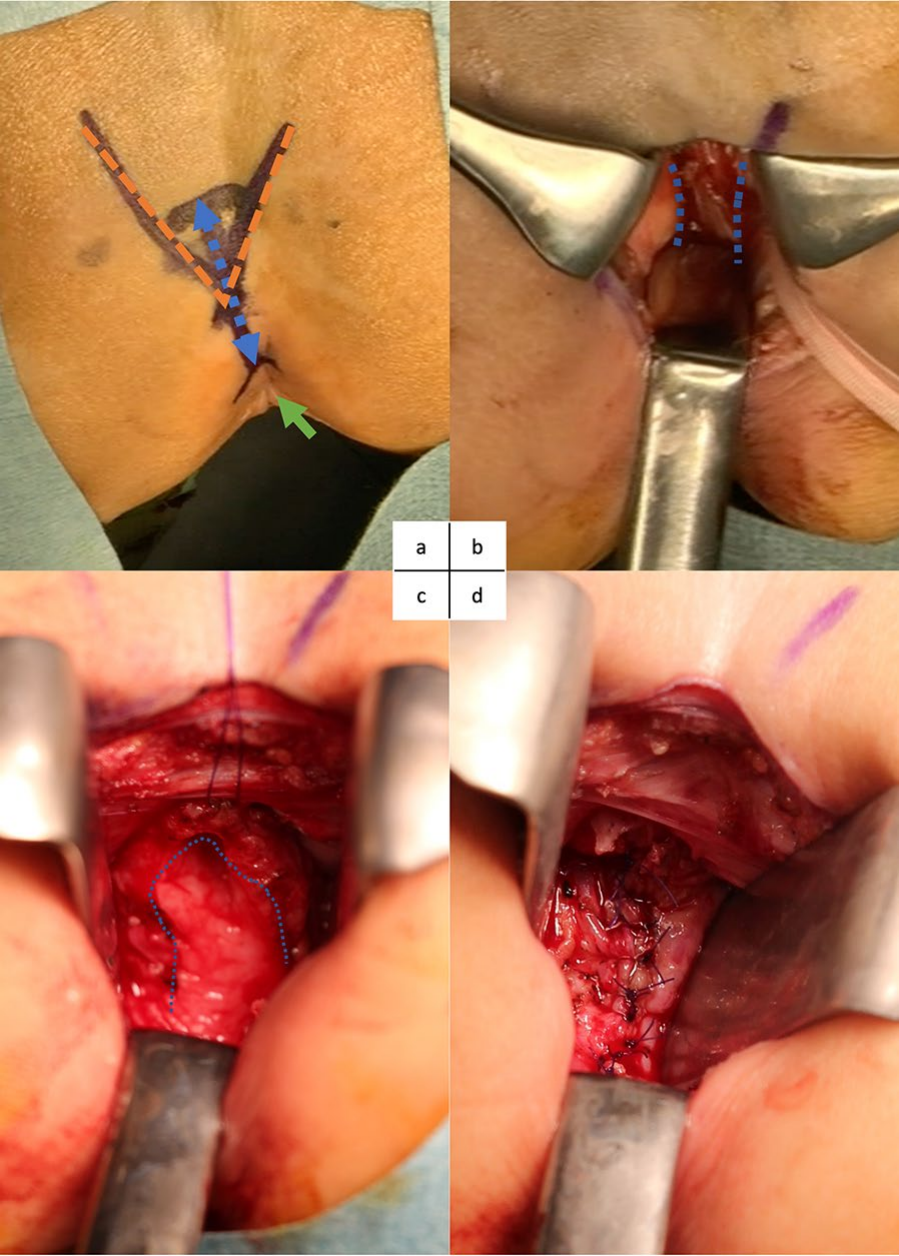
Images of the posterior sagittal approach. **a** An incision (double arrow) is placed in the midline between the anus (single arrow) and coccyx (break line), deep into the layers to approach the rectum. **b** The rectum is taped and pushed aside to the right. The lateral wall of the vagina is lined with dots. **c** The anterior wall of the vagina is exposed after resection of the tumor. The dotted line indicates the edge of the vaginal wall. **d** Reconstruction of the vagina

The patient was then placed in the jackknife position, and a 5-cm posterior sagittal incision was made (Fig. 6a); this approach is similar to posterior sagittal anorectoplasty (PSARP) for the correction of an imperforated anus [9]. The rectum was identified and taped after dissection through the subcutaneous and muscular layers (Fig. 6b). It was easy to separate the lateral side of the rectum because most of the separations had already been performed laparoscopically. The vagina and markers were easily identified by pushing the rectum aside, and the tumor was resected with direct visualization of the markers, vaginal wall, and tumor (Fig. 6c). After complete resection of the tumor from the vagina, we reconstructed the vagina with intermittent sutures on the posterior wall (Fig. 6d). A 14-Fr soft plastic tube was inserted to secure the cavity. The rectum was placed back, the dissected muscle and subcutaneous tissues were sutured in layers, and the incision was closed.

Pathological examination of the resected tumor confirmed complete resection of the tumor with viable tumor cells without invasion of the vertical or horizontal margins. After recovery from surgery, the patient received the third and fourth cycles of the ICE regimen as adjuvant therapy. AFP levels expectedly dropped and remained within the normal range (Fig. 2).

At each step of the treatment, the tumor board was held to discuss how to manage the patient including the regimen of chemotherapy and the surgical strategy. No complications of definitive surgery other than an episode of urinary tract infection, possibly due to temporary urinary incontinence, were observed.

## Discussion

Current evidence suggests that more than 80% of the patients with COG stage IV extracranial germ cell tumors survive. Additionally, the prognosis for vaginal YST is good [6]. Therefore, the next challenge for this tumor category is reducing the morbidity of treatment, chemotoxicity, surgical complications, and loss of organ functionality. Unlike the testis or ovary, the vagina is difficult to operate on because it resides deep in the pelvis. We speculate that this difficulty led to the idea of treating vaginal YST with chemotherapy alone, and recent reports supported the idea of chemotherapy being effective for many patients [6]. However, the tumor of this patient was resistant to chemotherapy and required surgical resection to control the tumor.

The development of a surgical strategy for vaginal YST began with total pelvic exenteration followed by vaginectomy with hysterectomy and bilateral salpingo-oophorectomy. However, these surgeries destroyed the reproductive system and were considered too invasive, especially after the cisplatin-based chemotherapy improved the prognosis of this tumor drastically. Thus, less invasive and organ-sparing surgery was considered to be ideal. The first successful preservation of childbearing potential was published in 1985 by Copeland [10], who reported that excisional biopsy followed by vincristine, actinomycin-D, and cyclophosphamide.

chemotherapy regimen was sufficient. Multiple favorable reports on the combination of chemotherapy and less invasive surgery have been published, and Mauz-Korholz reported the usefulness of this combination after nationwide multicenter clinical trials in Germany [11]. Organ-sparing surgery of the vagina could include trachelectomy and partial resection of the vagina, which can be performed trans-vaginally in adults. However, the pediatric vagina is too small to perform transvaginal surgery and leaves only a small amount of tissue after resection, making reconstruction difficult. Moreover, the transabdominal approach to the vagina is difficult because it extends from the surface of the perineum to the deepest part of the pelvis, where laparotomy provides only a small field of vision [12, 13, 13]. There are a few reports of cases where trachelectomy was adopted as a fertility-preserving approach for pediatric patients; however, that approach was determined to be too very invasive for this patient as the tumor was distant from the cervix and localized to the vaginal wall [12–14].

Our approach was based on anorectoplasty for the treatment of high-type imperforated anus. The laparoscope provides an enlarged view of the boundary of the vagina and rectum and makes it easy to detect infiltration of the tumor element into the adjacent tissue. Based on this view, it is also possible to separate the vagina and other structures in a minimally invasive manner, preserving anorectal and urinary function. Endoscopy of the vagina was also performed to confirm the perimeter of the tumor, particularly at both proximal and distal ends. Simultaneous investigation of the tumor from both inside and outside the vagina can ensure complete resection of the tumor.

However, the lateral and posterior sides of the vagina are difficult to investigate using a laparoscope. Our posterior sagittal approach gave us direct vision of the posterior and lateral walls of the vagina and thus made it easy to resect the tumor and reconstruct the posterior wall with good working space for sutures.

In hindsight, we may have relied excessively on chemotherapy to achieve complete remission of the tumor. Ideally, definitive surgery should have been performed a few cycles before the end of JEB; thus, we could have avoided the gonadotoxic ICE regimen. However, the decline of AFP was logarithmically linear, and we did not expect residual tumor to be present before the end of JEB. To date, there is no evidence of an approach to identify whether a tumor is chemo-resistant. However, repeated endoscopy and incisional biopsy may provide useful information. Accumulation of similar experiences is expected to provide additional insight into this topic.

## Conclusions

Herein, we report the usefulness of a novel approach for treating vaginal tumors. Our combination of laparoscopy, endoscopy, and posterior sagittal approach ensures a tumor-free macroscopic surgical margin with easier reconstruction of the vagina in a cosmetically beautiful manner while preserving the anorectal and urinary functions. We believe that this approach could be adopted for any vaginal tumor, especially those arising from the posterior or lateral wall.

## Data Availability

Not applicable.

## Abbreviations

YST: Yolk sac tumor
AFP: Alfa fetoprotein
GCT: Germ cell tumor
COG: Children’s Oncology Group
JEB: A regimen including carboplatin, etoposide, and bleomycin
ICE: A regimen including ifosfamide, carboplatin and etoposide
CT: Computed tomography
MRI: Magnetic resonance imaging
